# Methyl 4-amino-3-methyl­benzoate

**DOI:** 10.1107/S1600536808006223

**Published:** 2008-04-23

**Authors:** Xiang Li, Lian-Shan Yuan, Dan Wang, Shan Liu, Cheng Yao

**Affiliations:** a Department of Applied Chemistry, College of Sciences, Nanjing University of Technolgy, Xinmofan Road No. 5, Nanjing 210009, People’s Republic of China; b Bioengineering Department, Xuzhou Higher Vocational College of Bioengineering, Mine West Road, Xuzhou 221006, People’s Republic of China

## Abstract

In the mol­ecule of the title compound, C_9_H_11_NO_2_, the methyl C and amino N atoms bonded to the benzene ring lie in the ring plane. Intra­molecular C—H⋯O hydrogen bonding results in the formation of a five-membered planar ring, which is oriented at a dihedral angle of 2.73 (3)° with respect to the benzene ring, so they are nearly coplanar. In the crystal structure, inter­molecular N—H⋯O hydrogen bonds link the mol­ecules into chains elongated along the *c* axis and stacked along the *b* axis.

## Related literature

For related literature, see: Ries *et al.* (1993 ▶); Engeli *et al.* (2000 ▶); Kintscher *et al.* (2004 ▶); Goossens *et al.* (2003 ▶); Kurtz *et al.* (2004 ▶). For bond-length data, see: Allen *et al.* (1987 ▶).
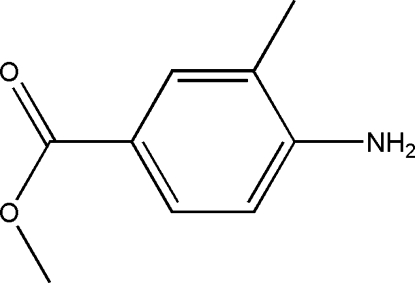


## Experimental

### 

#### Crystal data

C_9_H_11_NO_2_
*M* *_r_* = 165.19
Monoclinic, 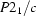

*a* = 7.5670 (15) Å
*b* = 6.1080 (12) Å
*c* = 18.127 (4) Å
β = 98.14 (3)°
*V* = 829.4 (3) Å^3^
*Z* = 4
Mo *K*α radiation
μ = 0.09 mm^−1^
*T* = 294 (2) K
0.40 × 0.30 × 0.20 mm

#### Data collection

Enraf–Nonius CAD-4 diffractometer
Absorption correction: ψ scan (North *et al.*, 1968 ▶) *T*_min_ = 0.963, *T*_max_ = 0.981
1747 measured reflections
1620 independent reflections
1079 reflections with *I* > 2σ(*I*)
*R*_int_ = 0.022
3 standard reflections every 200 reflections intensity decay: none

#### Refinement

*R*[*F* ^2^ > 2σ(*F* ^2^)] = 0.053
*wR*(*F* ^2^) = 0.188
*S* = 1.04
1620 reflections
109 parameters
H-atom parameters constrained
Δρ_max_ = 0.25 e Å^−3^
Δρ_min_ = −0.27 e Å^−3^

### 

Data collection: *CAD-4 Software* (Enraf–Nonius, 1985 ▶); cell refinement: *CAD-4 Software*; data reduction: *XCAD4* (Harms & Wocadlo, 1995 ▶); program(s) used to solve structure: *SHELXS97* (Sheldrick, 2008 ▶); program(s) used to refine structure: *SHELXL97* (Sheldrick, 2008 ▶); molecular graphics: *SHELXTL* (Sheldrick, 2008 ▶); software used to prepare material for publication: *SHELXTL*.

## Supplementary Material

Crystal structure: contains datablocks global, I. DOI: 10.1107/S1600536808006223/hk2431sup1.cif

Structure factors: contains datablocks I. DOI: 10.1107/S1600536808006223/hk2431Isup2.hkl

Additional supplementary materials:  crystallographic information; 3D view; checkCIF report

## Figures and Tables

**Table 1:** Hydrogen-bond geometry (Å, °)

*D*—H⋯*A*	*D*—H	H⋯*A*	*D*⋯*A*	*D*—H⋯*A*
C4—H4*A*⋯O1	0.93	2.40	2.728 (4)	100
N—H0*B*⋯O2^i^	0.86	2.37	3.142 (3)	150

Symmetry code: (i) 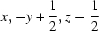.

**Fig. 1.**
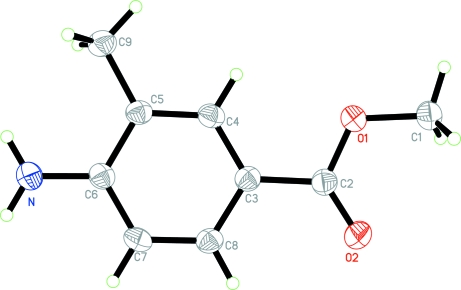
The molecular structure of the title molecule, with the atom-numbering scheme. Displacement ellipsoids are drawn at the 30% probability level.

**Fig. 2.**
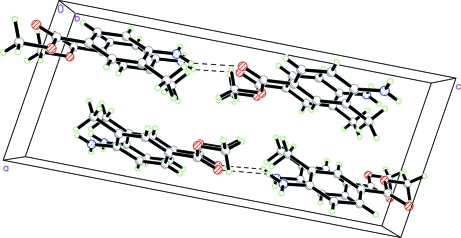
A packing diagram for (I). Hydrogen bonds are shown as dashed lines.

## supplementary crystallographic information

### Comment

Methyl 3-methyl-4-aminobenzoate is important as an intermedicine to prepare telmisartan, an angiotensin II receptor blocker, on the development of obesity and related metabolic disorders in diet-induced obese mice (Ries *et al.*, 1993). Telmisartan can be used as a therapeutic tool for metabolic syndrome, including visceral obesity (Engeli *et al.*, 2000; Kintscher *et al.*, 2004; Goossens *et al.*, 2003; Kurtz *et al.*, 2004). As part of our studies in this area, we report herein the synthesis and crystal structure of the title compound, (I).

In the molecule of (I), (Fig. 1), the ligand bond lengths (Allen *et al.*, 1987) and angles are within normal ranges. The atoms N and C9 lie in the benzene ring plane. The intramolecular C—H···O hydrogen bond (Table 1) results in the formation of a five-membered planar ring A (O1/C2/C3/C4/H4A), in which it is oriented with respect to the six-membered planar ring B (C3—C8) at a dihedral angle of A/B = 2.73 (3)°. So, they are also nearly coplanar.

In the crystal structure, intermolecular N—H···O hydrogen bonds (Table 1) link the molecules into chains elongated along the *c* axis and stacked along the *b* axis (Fig. 2), in which they may be effective in the stabilization of the structure.

### Experimental

The title compound (I) was prepared from 3-methyl-4-aminobenzoic acid (38 g, 250 mmol) in methanol (101 ml, 250 mmol). After the solid has melted, concentrated sulfuric acid (16 ml, 300 mmol) was dropped from the dropping funnel at 363 K, the latter was treated with a mixture of ice and water. The product was filtered by suction. Crystals of (I) suitable for X-ray diffraction were obtained by slow evaporation of an ethanol solution.

### Refinement

H atoms were positioned geometrically, with N—H = 0.86 Å (for NH_2_) and C—H = 0.93 and 0.96 Å for aromatic and methyl H, respectively, and constrained to ride on their parent atoms, with *U*_iso_(H) = *xU*_eq_(C,*N*), where *x* = 1.5 for methyl H, and *x* = 1.2 for all other H atoms.

### Crystal data

**Table:**

C_9_H_11_NO_2_	*F*_000_ = 352
*M**_r_* = 165.19	*D*_x_ = 1.323 Mg m^−^^3^
Monoclinic, *P*2_1_/*c*	Melting point: 391(2) K
Hall symbol: -P 2ybc	Mo *K*α radiation λ = 0.71073 Å
*a* = 7.5670 (15) Å	Cell parameters from 25 reflections
*b* = 6.1080 (12) Å	θ = 9–13º
*c* = 18.127 (4) Å	µ = 0.09 mm^−^^1^
β = 98.14 (3)º	*T* = 294 (2) K
*V* = 829.4 (3) Å^3^	Block, colorless
*Z* = 4	0.40 × 0.30 × 0.20 mm

### Data collection

**Table:**

Enraf–Nonius CAD-4 diffractometer	*R*_int_ = 0.022
Radiation source: fine-focus sealed tube	θ_max_ = 26.0º
Monochromator: graphite	θ_min_ = 2.3º
*T* = 294(2) K	*h* = −9→9
ω/2θ scans	*k* = 0→7
Absorption correction: ψ scan(North *et al.*, 1968)	*l* = 0→22
*T*_min_ = 0.963, *T*_max_ = 0.981	3 standard reflections
1747 measured reflections	every 200 reflections
1620 independent reflections	intensity decay: none
1079 reflections with *I* > 2σ(*I*)	

### Refinement

**Table:**

Refinement on *F*^2^	Secondary atom site location: difference Fourier map
Least-squares matrix: full	Hydrogen site location: inferred from neighbouring sites
*R*[*F*^2^ > 2σ(*F*^2^)] = 0.053	H-atom parameters constrained
*wR*(*F*^2^) = 0.188	*w* = 1/[σ^2^(*F*_o_^2^) + (0.06*P*)^2^ + 1.3*P*] where *P* = (*F*_o_^2^ + 2*F*_c_^2^)/3
*S* = 1.04	(Δ/σ)_max_ < 0.001
1620 reflections	Δρ_max_ = 0.25 e Å^−^^3^
109 parameters	Δρ_min_ = −0.27 e Å^−^^3^
Primary atom site location: structure-invariant direct methods	Extinction correction: none

### Special details

**Table:**

Geometry. All e.s.d.'s (except the e.s.d. in the dihedral angle between two l.s. planes) are estimated using the full covariance matrix. The cell e.s.d.'s are taken into account individually in the estimation of e.s.d.'s in distances, angles and torsion angles; correlations between e.s.d.'s in cell parameters are only used when they are defined by crystal symmetry. An approximate (isotropic) treatment of cell e.s.d.'s is used for estimating e.s.d.'s involving l.s. planes.
Refinement. Refinement of *F*^2^ against ALL reflections. The weighted *R*-factor *wR* and goodness of fit *S* are based on *F*^2^, conventional *R*-factors *R* are based on *F*, with *F* set to zero for negative *F*^2^. The threshold expression of *F*^2^ > σ(*F*^2^) is used only for calculating *R*-factors(gt) *etc*. and is not relevant to the choice of reflections for refinement. *R*-factors based on *F*^2^ are statistically about twice as large as those based on *F*, and *R*- factors based on ALL data will be even larger.

### Fractional atomic coordinates and isotropic or equivalent isotropic displacement parameters (Å^2^)

**Table:**

	*x*	*y*	*z*	*U*_iso_*/*U*_eq_	
N	0.8206 (4)	0.4908 (5)	0.17914 (15)	0.0570 (8)	
H0A	0.8716	0.6166	0.1792	0.068*	
H0B	0.7811	0.4279	0.1376	0.068*	
O1	0.6922 (3)	−0.1169 (4)	0.44629 (12)	0.0549 (7)	
O2	0.8163 (4)	0.1644 (4)	0.51362 (13)	0.0621 (8)	
C1	0.6852 (5)	−0.2393 (6)	0.51341 (18)	0.0561 (10)	
H1A	0.6303	−0.3788	0.5012	0.084*	
H1B	0.6164	−0.1599	0.5451	0.084*	
H1C	0.8041	−0.2610	0.5388	0.084*	
C2	0.7641 (4)	0.0832 (5)	0.45389 (18)	0.0440 (8)	
C3	0.7726 (4)	0.1877 (5)	0.38130 (17)	0.0404 (7)	
C4	0.7052 (4)	0.0892 (5)	0.31364 (17)	0.0423 (8)	
H4A	0.6496	−0.0464	0.3145	0.051*	
C5	0.7176 (4)	0.1847 (5)	0.24532 (17)	0.0405 (8)	
C6	0.8021 (4)	0.3903 (5)	0.24530 (17)	0.0414 (7)	
C7	0.8662 (4)	0.4927 (5)	0.31251 (18)	0.0450 (8)	
H7A	0.9196	0.6297	0.3120	0.054*	
C8	0.8517 (4)	0.3940 (5)	0.37935 (18)	0.0427 (8)	
H8A	0.8947	0.4649	0.4237	0.051*	
C9	0.6433 (5)	0.0725 (6)	0.17397 (18)	0.0525 (9)	
H9A	0.5906	−0.0644	0.1851	0.079*	
H9B	0.7377	0.0463	0.1448	0.079*	
H9C	0.5541	0.1639	0.1464	0.079*	

### Atomic displacement parameters (Å^2^)

**Table:**

	*U*^11^	*U*^22^	*U*^33^	*U*^12^	*U*^13^	*U*^23^
N	0.076 (2)	0.0464 (17)	0.0479 (17)	−0.0106 (16)	0.0050 (15)	0.0071 (14)
O1	0.0748 (17)	0.0429 (13)	0.0455 (13)	−0.0097 (13)	0.0031 (11)	0.0032 (11)
O2	0.090 (2)	0.0538 (16)	0.0409 (13)	−0.0084 (14)	0.0053 (13)	−0.0040 (12)
C1	0.076 (3)	0.048 (2)	0.0449 (19)	−0.0029 (19)	0.0100 (17)	0.0037 (16)
C2	0.0489 (19)	0.0416 (18)	0.0418 (17)	0.0019 (16)	0.0079 (14)	−0.0005 (15)
C3	0.0425 (17)	0.0373 (17)	0.0414 (17)	0.0040 (14)	0.0058 (13)	−0.0029 (14)
C4	0.0430 (18)	0.0349 (17)	0.0479 (18)	−0.0003 (14)	0.0021 (14)	−0.0008 (14)
C5	0.0419 (18)	0.0344 (16)	0.0435 (17)	0.0038 (14)	0.0003 (13)	−0.0020 (14)
C6	0.0448 (18)	0.0325 (16)	0.0468 (17)	0.0050 (14)	0.0065 (14)	0.0022 (14)
C7	0.0458 (19)	0.0327 (16)	0.056 (2)	−0.0036 (14)	0.0061 (15)	−0.0014 (15)
C8	0.0477 (18)	0.0372 (17)	0.0434 (17)	0.0030 (15)	0.0071 (13)	−0.0067 (14)
C9	0.057 (2)	0.051 (2)	0.0469 (19)	−0.0055 (17)	−0.0031 (16)	−0.0022 (16)

### Geometric parameters (Å, °)

**Table:**

N—C6	1.372 (4)	C4—C5	1.384 (4)
N—H0A	0.8600	C4—H4A	0.9300
N—H0B	0.8600	C5—C6	1.410 (4)
O1—C2	1.336 (4)	C5—C9	1.501 (4)
O1—C1	1.435 (4)	C6—C7	1.394 (4)
C1—H1A	0.9600	C7—C8	1.372 (4)
C1—H1B	0.9600	C7—H7A	0.9300
C1—H1C	0.9600	C8—H8A	0.9300
O2—C2	1.206 (4)	C9—H9A	0.9600
C2—C3	1.472 (4)	C9—H9B	0.9600
C3—C4	1.396 (4)	C9—H9C	0.9600
C3—C8	1.398 (4)		
			
C6—N—H0A	120.0	C4—C5—C6	117.6 (3)
C6—N—H0B	120.0	C4—C5—C9	120.9 (3)
H0A—N—H0B	120.0	C6—C5—C9	121.4 (3)
C2—O1—C1	116.9 (3)	N—C6—C7	119.9 (3)
O1—C1—H1A	109.5	N—C6—C5	120.1 (3)
O1—C1—H1B	109.5	C7—C6—C5	120.1 (3)
H1A—C1—H1B	109.5	C8—C7—C6	120.9 (3)
O1—C1—H1C	109.5	C8—C7—H7A	119.5
H1A—C1—H1C	109.5	C6—C7—H7A	119.5
H1B—C1—H1C	109.5	C7—C8—C3	120.4 (3)
O2—C2—O1	123.1 (3)	C7—C8—H8A	119.8
O2—C2—C3	125.0 (3)	C3—C8—H8A	119.8
O1—C2—C3	111.9 (3)	C5—C9—H9A	109.5
C4—C3—C8	118.1 (3)	C5—C9—H9B	109.5
C4—C3—C2	122.8 (3)	H9A—C9—H9B	109.5
C8—C3—C2	119.1 (3)	C5—C9—H9C	109.5
C5—C4—C3	122.8 (3)	H9A—C9—H9C	109.5
C5—C4—H4A	118.6	H9B—C9—H9C	109.5
C3—C4—H4A	118.6		
			
C1—O1—C2—O2	−2.4 (5)	C4—C5—C6—N	179.0 (3)
C1—O1—C2—C3	177.0 (3)	C9—C5—C6—N	−1.1 (5)
O2—C2—C3—C4	−178.0 (3)	C4—C5—C6—C7	−1.4 (4)
O1—C2—C3—C4	2.7 (4)	C9—C5—C6—C7	178.5 (3)
O2—C2—C3—C8	2.2 (5)	N—C6—C7—C8	−179.0 (3)
O1—C2—C3—C8	−177.2 (3)	C5—C6—C7—C8	1.3 (5)
C8—C3—C4—C5	1.7 (5)	C6—C7—C8—C3	0.3 (5)
C2—C3—C4—C5	−178.2 (3)	C4—C3—C8—C7	−1.7 (5)
C3—C4—C5—C6	−0.1 (5)	C2—C3—C8—C7	178.1 (3)
C3—C4—C5—C9	180.0 (3)		

### Hydrogen-bond geometry (Å, °)

**Table:**

*D*—H···*A*	*D*—H	H···*A*	*D*···*A*	*D*—H···*A*
C4—H4A···O1	0.93	2.40	2.728 (4)	100
N—H0B···O2^i^	0.86	2.37	3.142 (3)	150

Symmetry codes: (i) *x*, −*y*+1/2, *z*−1/2.
